# Predicting the Conversion from Mild Cognitive Impairment to Alzheimer’s Disease Using Graph Frequency Bands and Functional Connectivity-Based Features

**DOI:** 10.21203/rs.3.rs-4549428/v1

**Published:** 2024-06-21

**Authors:** Jafar Zamani, Alireza Talesh Jafadideh

**Affiliations:** 1 Department of Psychiatry and Behavioral Sciences, Stanford University, California, USA; 2 School of Engineering Science, College of Engineering, University of Tehran, Tehran, Iran.

**Keywords:** Mild cognitive impairment, Alzheimer’s disease, graph signal processing, connectivity-based features, classification

## Abstract

Accurate prediction of the progression from mild cognitive impairment (MCI) to Alzheimer’s disease (AD) is crucial for disease management. Machine learning techniques have demonstrated success in classifying AD and MCI cases, particularly with the use of resting-state functional magnetic resonance imaging (rs-fMRI) data.This study utilized three years of rs-fMRI data from the ADNI, involving 142 patients with stable MCI (sMCI) and 136 with progressive MCI (pMCI). Graph signal processing was applied to filter rs-fMRI data into low, middle, and high frequency bands. Connectivity-based features were derived from both filtered and unfiltered data, resulting in a comprehensive set of 100 features, including global graph metrics, minimum spanning tree (MST) metrics, triadic interaction metrics, hub tendency metrics, and the number of links. Feature selection was enhanced using particle swarm optimization (PSO) and simulated annealing (SA). A support vector machine (SVM) with a radial basis function (RBF) kernel and a 10-fold cross-validation setup were employed for classification. The proposed approach demonstrated superior performance, achieving optimal accuracy with minimal feature utilization. When PSO selected five features, SVM exhibited accuracy, specificity, and sensitivity rates of 77%, 70%, and 83%, respectively. The identified features were as follows: (Mean of clustering coefficient, Mean of strength)/Radius/(Mean Eccentricity, and Modularity) from low/middle/high frequency bands of graph. The study highlights the efficacy of the proposed framework in identifying individuals at risk of AD development using a parsimonious feature set. This approach holds promise for advancing the precision of MCI to AD progression prediction, aiding in early diagnosis and intervention strategies.

## Introduction

Dementia affects approximately 50 million individuals worldwide, with nearly 10 million new cases emerging annually[1]. Among dementia subtypes, Alzheimer’s disease (AD) represents the most prevalent form, accounting for over half of all cases. Amnestic mild cognitive impairment (MCI) occupies a pivotal intermediate stage between healthy controls (HC) and AD. Individuals with MCI face an escalated risk of transitioning to AD, with an approximate annual conversion rate of 15%. Notably, the MCI cohort exhibits significant heterogeneity, with only a subset progressing to AD [1,2]. Early-stage intervention for AD poses a considerable clinical challenge. Established biomarkers for AD prediction include the accumulation of Aβ and hyperphosphorylated tau [3]. Verification of amyloid and tau deposits traditionally necessitates invasive techniques such as positron emission tomography (PET) and cerebrospinal fluid (CSF) analysis. Conversely, magnetic resonance imaging (MRI) enables the evaluation of neurodegenerative signs, including atrophy and neuronal loss, indicative of amyloid and tau deposition [4].

MRI and resting-state functional MRI (rs-fMRI) has emerged as a supportive tool for the early-stage clinical assessment of AD or disease progression. While task-based fMRI examines brain function during cognitive tasks, rs-fMRI captures spontaneous low-frequency brain activity, making it valuable for AD diagnosis [5,6]. In conventional functional connectivity (FC) analyses, brain region correlations are assumed to remain constant throughout an imaging session. Dynamic FC, a more recent extension of traditional FC, captures evolving interactions and is considered a more accurate representation of functional brain networks [6,7].

Robust biomarker identification is pivotal for distinguishing progressive MCI (pMCI) from stable MCI (sMCI), facilitating early AD diagnosis and treatment. PMCI refers to individuals with MCI who exhibit a continuous decline in cognitive function, ultimately progressing to AD. SMCI refers to individuals whose cognitive impairment does not significantly worsen over time, remaining stable without advancing to AD. Recent endeavors have integrated multimodal biomarkers, including PET and rs-fMRI, with machine learning algorithms for predicting the conversion from MCI to AD [9–12]. Notably, functional neuroimaging holds greater promise for early AD detection compared to structural neuroimaging [13–15]. Functional MRI, which evaluates brain function during cognitive tasks, demonstrates remarkable sensitivity to early disease processes, often preceding observable impairments in standard neuropsychological tests [16,17]. Conversely, rs-fMRI captures spontaneous brain activity fluctuations, making it less dependent on individual cognitive capabilities [18–20].

Key among rs-fMRI’s attributes is its capacity to assess functional connectivity (FC) alterations [21,22], a prevalent hallmark of AD [23–26]. Studies have shown that cognitive impairment severity correlates with increasing disruptions in connectivity patterns, suggesting FC changes as potential cognitive dysfunction biomarkers in MCI. Importantly, longitudinal FC alterations are more pronounced in early AD stages [27]. FC analysis inherently involves network interactions, making graph theory an effective tool for investigating global and local brain region characteristics [28–31]. This approach has successfully elucidated insights into various neurological conditions, including depression, Parkinson’s disease, and AD. This method has been used successfully in a wide range of applications in both healthy participants and patients [32], such as depression [33,34], Parkinson’s disease [35], as well as AD [36]. Graph theory, a powerful topological tool, allows us to investigate AD in new ways [36–39]. It enables us to compare the brain network organization between patients and healthy individuals [40,41], and importantly, provides insights into how these networks change across different stages of the disease [42,43]. This method delves deeper, not only identifying brain differences but also revealing compensatory mechanisms that might explain why some individuals with similar cognitive scores exhibit different brain activity patterns [44–47].

Graph theory methods such as the minimum spanning tree (MST) provide valuable insights into brain connectivity. In this context, nodes represent brain regions, and edges represent the functional connections (weights) between these regions. The MST is a subgraph that connects all nodes with the minimum possible total edge weight, avoiding cycles and redundant connections. This simplification retains essential network structure, offering an “impartial” representation by focusing on the most critical connections. This impartial technique significantly streamlines the network structure while retaining its essential framework. Notably, it ensures the neurological interpretability of the network, making it a widely employed tool in neuroimaging [48,49]. Through this method, the edges within the network undergo simplification, ensuring that the selected spanning tree possesses the smallest conceivable weight.

While most brain network analyses focus on pairwise interactions between regions, the complex reality of the human brain suggests higher-order interactions play a crucial role. Investigating higher-order interactions within the brain network can lead to groundbreaking discoveries related to brain function and dysfunction, disease progression, and potentially, treatment development. Moradimanesh et al. [50] delved deeper into brain network analysis by examining triadic interactions, involving three interconnected regions. This method allowed them to compare the interaction patterns between individuals with autism spectrum disorder (ASD) and HC. Pearson correlation served as their tool to measure the interaction between regions. The authors explored four distinct triadic interaction patterns, each with specific configurations of positive and negative FC values (+ and −). These triads were strongly balanced T_3_ : (+ + +), strongly unbalanced T_2_ : (+ + −), weakly balanced T_1_ : (+ − −), and weakly unbalanced T_0_ : (− − −). The study revealed that the balanced brain interactions were more common in both ASD and HC groups, while unbalanced interactions were less frequent. Additionally, the energy levels of the salience network (SN) and the default mode network (DMN), were found to be lower in AD patients, suggesting potential challenges in adapting behavior. In another study of triadic interactions, Saberi and colleagues introduced the metrics of *tendency to make hub* (TMH) and showed that negative links of the resting-state network make hubs to reduce balance-energy and push the network into a more stable state compared to null-networks with trivial topologies [51].

Graph signal processing (GSP) is a recently developed field that analyzes brain activity through a unique lens called the topological frquency [52–57]. This approach relies on two key elements: a graph representing brain connections and brain activity itself mapped onto that graph. Using a tool called the graph Fourier transform (GFT), GSP can compute different topological frequency filters and, consequently, identify different patterns hidden within these connections. Excitingly, recent research has shown that GSP can be used to diagnose early-stage Mild Cognitive Impairment (MCI) based on brain activity data from two independent studies [58,59].

Early diagnosis of AD at the MCI stage is very important for the development of efficacy treatments. However, the heterogeneity of AD has made early diagnosis a challenging problem. Many machine learning algorithms have been applied for the diagnosis of MCI and predicting MCI to AD conversion [60,61]. Because of the huge number of extracted features from the neuroimaging data, a feature selection step is extremely important before classification. Modern machine learning methods often incorporate implicit feature selection mechanisms. While explicit feature selection as a preprocessing step is less common, it remains beneficial for reducing dataset dimensionality and improving classification accuracy. By performing this step, the most representative optimal feature set is selected and the redundant features for the diagnosis of AD progression are neglected [62,63]. The high-efficacy feature selection algorithms are useful to speed up the diagnostic system and enhance its diagnostic performance. The performance of feature selection and classification methods depends on the tuning of hyperparameters and the specific characteristics of the dataset. Effective optimization requires careful consideration of these parameters to achieve robust results. Feature selection is particularly complicated due to the nonlinear nature of classification methods: more parameters do not necessarily lead to better performance, and there is also a dependency on parameters. Therefore, it is extremely important to utilize a suitable optimization method that can deal with nonlinear high-dimensional search spaces [64,65].

In this study, the topological filters were obtained through GFT tool and sparse FC (SFC) matrix. Each subject possessed a unique SFC matrix, computed through Pearson correlation and the Wilcoxon rank sum test [66]. The GFT was used to compute three topological frequency filters which were then used to separate the brain activity data (rs-fMRI) into three distinct frequency bands: low, middle, and high (abbreviated as LFB, MFB, and HFB, respectively). FC matrices were computed for each aforementioned frequency band using the filtered data. Additionally, an FC matrix was computed for the unfiltered data, termed the full-frequency band (FFB). Some of the graph global metrics, MST metrics, triadic interaction metrics, TMH metrics, and the number of positive and negative links were computed from LFB, MFB, HFB, and FFB FC matrices. To obtain the most important features, the feature selection was carried out using particle swarm optimization (PSO) and simulated annealing (SA) [67,68]. Subsequently, the selected features were employed for the classification of AD and MCI. Despite achieving superior accuracy compared to numerous prior methods, our analysis relied on a limited number of fMRI features, resulting in lower computational complexity than multi-modality data approaches. Our analysis offers features based on FC, which are easy to interpret and understand.

The rest of the paper is organized as follows: Section 2 describes the dataset, preprocessing methods, brain parcellation, FC, graph frequency bands, studied features, feature selection, and classification are explained in the Material and methods section. The subsequent section, Results, presents the outcomes of the feature selection and classification processes. Lastly, the following two sections engage in a discussion of the results and present the concluding remarks and insights.

## Materials and methods

### Participants and data acquisition

In this study, data for a total of 278 human participants were used. These human participants data were extracted from the Alzheimer’s disease Neuroimaging Initiative database (ADNI) [69,70] (Demographics information are reported in Table 1; The mini-mental state exam (MMSE) is a cognitive measure that is widely used in clinical and research settings to measure the cognitive status of AD). The studied data (ADNI’s data) can be accessed at http://adni.loni.usc.edu/. Other researchers can access to this data using the same procedures as the authors did. Researchers can access the data by logging into the ADNI website and following these steps: Download > Image Collections > Advanced Search > Search > Select the scans > Add to collection > CSV download > Advanced download. A complete listing of ADNI investigators can be found at: http://adni.loni.usc.edu/study-design/ongoing-investigations/. The public access to the database is open. The ADNI was launched in 2003 to test whether serial MRI, fMRI, other biological markers, and clinical and neuropsychological assessments can be combined to measure the progression of MCI and early Alzheimer’s disease (AD). In the subjects selected for this study, we use a subject with at least three years of follow-up diagnosed with MCI at the baseline evaluation. The selected subjects with fixed Clinical Dementia Rating (CDR) scores of 0.5 in the follow-up period are sMCI, and the CDR of the pMCI in the baseline and final are 0.5 and 1, respectively [12]. The studied rs-fMRI data had been measured using a high field 3-Tesla Philips MRI scan machin and an echo-planar imaging technique. Data of eachsubject consisted of 140 volumes each with 48 slices, 3.3mm slice thickness, spatial resolution of 3×3×3 mm^3^, flip angle of 80 degree, 30ms echo time, and plane matrix of 64×64. The time between two consecutive volumes was 2s.

### Data preprocessing

#### rs-fMRI data preprocessing and time series extraction

The preprocessing pipeline for the rs-fMRI data comprised several essential steps to ensure data quality and reliability. The initial five volumes were discarded to mitigate the influence of T1-equilibration effects. Subsequent preprocessing steps encompassed functional realignment and unwarping, correction for slice-timing discrepancies, identification, and handling of outlier volumes to address subject-motion artifacts, direct segmentation, and normalization into the standard MNI space, and spatial convolution with an 8mm full-width half-maximum Gaussian kernel for functional smoothing. Low-frequency filtering within the range of 0.01 to 0.1 Hz was applied to retain the relevant fluctuations [71].

The preprocessing of rs-fMRI data was executed using the CONN toolbox. The Harvard-Oxford Cortical atlas with 136 regions of interest (ROIs) was employed for brain parcellation. For each ROI, one signal was obtained by averaging the time series of that ROI voxels. The final rfMRI data was $x\in {\mathbb{R}}^{M\times T}$ where M = 136 and T = 135 were the numbers of ROIs and time samples, respectively.

#### Graph frequency bands (GFBs)

**Fig 1.**
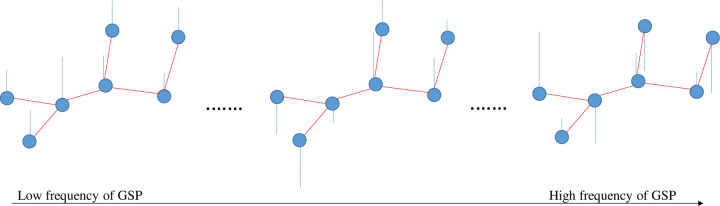
Simple representation of frequency concept in graph domain. In this domain, the signal changes across connected vertices define frequency levels (in the time domain, the signal changes across time points define frequency levels). Consequently, transitioning from lower to higher frequency levels within the graph amplifies the signal changes across connected vertices. Blue circles and red and blue lines are vertices, edges, and signals, respectively.

The frequency content of the graph signal is defined according to the signal changes across connected vertices at a given time point. In low frequency, connected vertices show similar signals (representing alignment). In high frequency, the variability of the connected vertices signals is high compared to each other (representing liberality). In liberality, the vertices (brain ROIs) show less respect for their underlying connectivity structure. By approaching from low frequency to high frequency, the graph signal behavior changes from alignment to liberality (Fig 1).

The graph frequencies are defined using the combinatorial Laplacian matrix $\text{L}\in {\mathbb{R}}^{N\times N}$, [52], as follows:

(1) L=D−A

where ***A*** is the adjacency matrix and **D** is a diagonal matrix, and its *k*^th^ diagonal element represents the degree of *k*^th^ vertex i.e., ${\text{D}}_{kk}=\sum_{j=1}^{N}{\text{A}}_{kj}$. The adjacency matrix represents the underlying graph of GSP. The eigendecomposition of **L** provides the **V** and **Λ**, which are the eigenvector matrix and diagonal eigenvalues matrix, respectively.

The eigenvectors represent graph frequency modes and are used for GFT. The GFT of brain signal $x\in {\mathbb{R}}^{136\times 135}$ is obtained as

(2) $$\tilde{x}=V^{T}x.$$

where 136 and 135 are the number of ROIs and time points and superscript *T* denotes the transpose operation, respectively. The inverse GFT (IGFT) of $\tilde{x}$ is attained by

(3) $$x=V\tilde{x}.$$

Remarkably, the eigenvector associated with the larger eigenvalue exhibits greater variance and can effectively convey higher graph frequencies [72]. These higher frequency modes facilitate the conversion of brain signals characterized by increased variance into the graph frequency domain. Conversely, they can also transform higher frequency information from the frequency domain back to the brain’s topological domain.

The graph signal is amenable to filtering within the frequency domain, followed by an IGFT to obtain a graph-filtered signal. The graph filtering process can be mathematically formulated as follows:

(4) $$x_{F}=VGV^{T}x$$

where **G** is a diagonal filtering matrix. In this study, a value of 1 was assigned to the diagonal elements corresponding to the desired frequency modes, while the rest of the modes were set to 0.

In this study, the LFB consisted of the first 45 frequency modes, the HFB comprised the last 45 modes, and the MFB was formed by the remaining 46 modes. Using the “(4)”, the rs-fMRI data underwent filtering to generate graphs corresponding to LFB, MFB, and HFB. Subsequently, for each subject, FC matrices were computed within the LFB, MFB, HFB, and FFB.In this study, the size of the data matrices for LFB, MFB, HFB, and FFB were 136 × 135.

### FC matrix

FC between ROIs was computed using Pearson correlation and the SW technique. The SW technique was employed to consider the dynamic nature of brain FC. In this approach, a series of windows with a one-TR shift was applied to each ROI time series. Subsequently, an FC matrix was computed for each window. The final correlation value for an ROI-ROI pair was determined as the median of its FC values. The window was created by convolving a rectangle (width = 50 TRs) with a Gaussian (σ = 3 TRs) [73]. Each subject’s dataset yielded four FC matrices. These matrices were computed using LFB, MFB, HFB, and FFB rs-fMRI data.

To attain the adjacency matrix (*A*) of GSP, the FC matrix of FFB was compared between sMCI and pMCI groups using the Wilcoxon rank sum test. This process identified statistically significant ROI-ROI connections. Subsequently, for each subject, these significant ROI-ROI connections from the FFB were retained, while the remaining connections were set to zero. Thus, for each subject, a sparse FC (SFC) matrix was computed using the FFB FC and the rank sum test. This matrix served as the adjacency matrix (A) for GSP.

### Features

Graph, MST, and triadic interaction metrics were individually computed for each of the four FC matrices. The features for this study were extracted from the data of 142 subjects with sMCI and 136 subjects with pMCI. The dimension of each FC matrix was 136 × 136 for the Harvard-Oxford atlas.

#### Global metrics of graph

A graph G is defined as a set of vertices V(G) and edges E(G). The connectivity matrix can be represented as a graph, where the ROIs serve as vertices, and the connectivity strengths act as the weights of the edges. This modeling approach facilitated the exploration of topological distinctions between ASD and TC groups using graph metrics. Subsequently, some of the graph global metrics are outlined below[74].

##### Global efficiency (GE):

GE) is defined as the average inverse shortest path length in the network. In this study, the shortest path between two ROIs is defined as the is the distance between them.

##### Mean Eccentricity (ME):

For each ROI, the eccentricity is equal to the maximum distance between that ROI and the rest of ROIs. ME equals the average eccentricity of all ROIs.

##### Radius:

The minimum value of eccentricity of all ROIs is equal to the radius.

##### Diameter:

The maximum value of eccentricity of all ROIs is equal to the diameter.

##### Assortativity coefficient (AC):

Each connection involves two ROIs: one initiating the connection and the other concluding it. Let’s denote the degrees of the first and second ROIs as x and y, respectively. Consequently, for all available connections, two vectors, X and Y, are obtained, with the first representing a set of degrees x and the second a set of degrees y. By calculating the correlation coefficient between X and Y, the value of the AC is derived. This coefficient ranges between −1 and 1, where positive values indicate that ROIs with similar degrees are inclined to connect. Conversely, a negative AC value implies that ROIs with larger degrees tend to connect to ROIs with smaller degrees.

##### Mean of clustering coefficient (MCC):

The Clustering Coefficient is the ratio of triangles around a ROI and ranges between 0 and 1. A value of 1 indicates that connected ROIs to a given ROI are also connected to each other. A lower number of connections in the vicinity of a given ROI result in a decreased clustering coefficient. In this study, the mean clustering coefficient values across all ROIs were calculated for each subject.

##### Mean of eigenvector centrality (MEC):

Connections originating from high-scoring ROIs carry more weight in influencing the score of the ROI under consideration compared to connections from low-scoring ROIs. The EC of an ROI reflects its impact on the network, where an ROI with high EC tends to connect with ROIs that also have high scores. For each subject in this study, the mean EC values across all ROIs were taken into consideration.

##### Mean of strength (MS):

The strength of a specific ROI is defined as the sum of weights of edges adjacent to that ROI. In this study, the mean strength values across all ROIs were calculated for each subject.

##### Modularity:

This metric gauges how effectively a network has been partitioned into groups of ROIs. In a network with high modularity, dense connections are observed within the groups, and there are sparse connections between the groups of ROIs.

All these metrics were computed using the functions provided by the Brain Connectivity Toolbox [75].

#### Metrics of minimum spanning tree

A spanning tree is a sub-graph of the original graph that is devoid of cycles or loops and connects all nodes in the original graph. The Minimum Spanning Tree (MST) is a tree with the minimum total weight among all possible spanning trees of the original graph [76,77]. In this study, the Single Linkage Dendrogram method was employed for the computation of the MST [78]. Hereafter, several metrics related to the MST are delineated. [77–79].

##### Radius and Diameter:

The minimum and maximum values of eccentricity for all ROIs in the MST correspond to the radius and diameter, respectively.

##### Maximum degree (Deg_max_):

The degree *k*_*i*_ is the number of neighbors for *i*^*th*^ ROI in the MST. The maximum of all ROI degrees is considered as Deg_max_.

##### Leaf fraction (LF):

The fraction of leaf ROIs in the MST, where a leaf ROI is defined as an ROI with a degree of one.

##### Maximum betweenness centrality (BC_max_):

The BC of a particular ROI is the fraction of all shortest paths that traverse through that ROI. The maximum value among all ROI BCs is considered as BC_max_.

##### Hierarchy (T_H_):

The tree hierarchy assesses the balance between large-scale integration in the MST, quantified by the leaf fraction, and the concentration of central nodes, also referred to as hubs, measured through the maximum BC. This metric can be expressed as

(5) $$T_{H}=LF/2BC_{max}$$

##### Kappa:

This metric quantifies the breadth of the degree distribution. This metric can be formulated as

(6) $$\text{Kappa}\;=\sum_{i=1}^{136}k_{i}^{2}/\sum_{i=1}^{136}k_{i}$$

#### Metrics of triadic interactions

**Fig 2.**
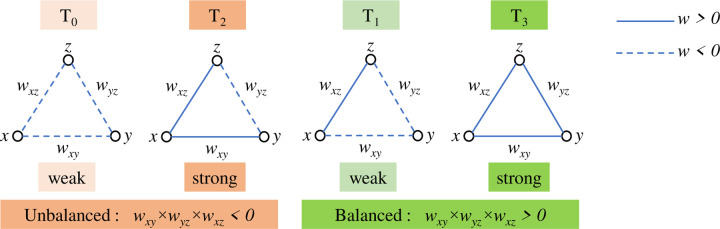
Four types of triads. The subscript of each T denotes the number of positive links.

In this study, as with Moradimanesh and colleagues [50], four types of triads were analyzed in LFB, MFB, HFB, and FFB. These triads were strongly balanced T_3_ : (+ + +), weakly balanced T_1_ : (+ −−), strongly unbalanced T_2_ : (+ + −), and weakly unbalanced T_0_ : (−−−) (Fig 2). Five metrics were extracted from FC matrices. The first four metrics were the number of the triads T_0_, T_1_, T_2_, and T_3_. These metrics are also called the frequency of triads (|T_i_|, i=0,1,2,3). The fifth one was the energy of the whole-brain network (*Un*). The *Un* is defined as

(7) $$Un=-\sum_{i=0}^{i=3}\sum_{x<y<z}w_{xy}\left(T_{i}\right)w_{xz}\left(T_{i}\right)w_{yz}\left(T_{i}\right)/\Delta$$

where x, y, and z indicate the ROIs of triad T_i_, *w* is the FC value between ROIs, and Δ is the total number of triads of the brain.

#### Tendency to make hub

Hubs are ROIs with a high number of connections and play a pivotal role in the topology of the brain network. In this study, we employed the global hubness metric introduced by Saberi and colleagues to examine the brain topology of healthy control subjects [51]. This metric, which is named the TMH, is separately defined for positive and negative links as *TMH*_*P*_ and *TMH*_*N*_:

(8) $$TMH_{P}=\sum_{i=1}^{136}D_{i,p}^{2}/\sum_{i=1}^{136}D_{i,p},D_{i,p}=\sum_{j=1,j\neq i,w_{ij}>0}^{136}w_{ij,p}$$

and

(9) $$TMH_{N}=\sum_{i=1}^{136}D_{i,n}^{2}/\sum_{i=1}^{136}D_{i,n},D_{i,n}=\sum_{j=1,j\neq i,w_{ij}<0}^{136}w_{ij,n}$$

where *136* is the total number of ROIs, *D*_*i,p*_ and *D*_*i,n*_ represent the positive and negative degrees of i^th^ ROI, respectively, and *w*_*ij,p*_ and *w*_*ij,n*_ are the positive and negative weights between i^th^ and j^th^ ROIs.

The *TMH*_*P*_ and *TMH*_*N*_ demonstrate the network’s propensity to form hubs with positive and negative links, respectively. Therefore, TMH can elucidate the influence of both positive and negative links on the topology of the brain.

#### The number of links

The number (or occurrence rate) of positive links |P| and negative links |N| are computed for FC matrices of LFB, MFB, HFB, and FFB, separately. By obtaining the information on |P| and |N|, it can be determined whether the |T_i_|s and TMHs may vary between groups even when there is no difference in the number of positive and negative links.

### Feature selection

A total of 100 features were extracted from the four FC matrices (LFB, MFB, HFB, FFB) for each subject. This set included 25 features in each matrix, distributed as follows: Graph (9), MST (7), Triadic (5), TMH (2), and Number of Links (2). To improve the efficiency and accuracy of the classification algorithm, a critical step was undertaken - feature selection.

Feature selection plays a pivotal role in machine learning by reducing dataset dimensionality and improving classification algorithm performance and accuracy. In this study, we employed two optimization algorithms, PSO and SA, to identify the most informative set of features [67,68].

PSO is a stochastic optimization technique inspired by the behavior of swarming animals like birds and fish. It operates by representing potential solutions as particles that traverse the search space. Particles adjust their positions and velocities based on cognitive and social parameters, and the overall rate of change is regulated by an inertia parameter. Specifically, particles seek optimal regions of the search space through interaction with other particles in the population. For our study, we utilized a swarm size of 20 particles, while setting cognitive and social parameters to 1.5 and inertia to 0.72.

SA employs a probabilistic approach to accept or reject solutions. The algorithm initiates with a randomly generated solution and iteratively generates neighboring solutions based on a predefined neighborhood structure. A fitness function evaluates each generated solution. Improved solutions are accepted, and worse neighbors are accepted probabilistically, governed by the Boltzmann probability equation, *P* = *e* − *θ*/ *T*. In this equation, *θ* denotes the difference between the fitness of the best solution and the generated neighbor, while *T* represents a temperature parameter. The temperature *T* decreases over iterations according to a cooling schedule. In our study, the initial temperature *T* was set to 10 [68].

By utilizing PSO and SA, we aimed to discern the optimal subset of features that significantly contribute to the classification task. This feature selection process not only streamlines the dataset but also enhances the classification performance, making our analysis more effective and efficient. Feature selection was performed within each fold of the cross-validation process to avoid test-set contamination and ensure unbiased evaluation of predictive performance.

## Classification

To discriminate between progressive mild cognitive impairment (pMCI) and stable mild cognitive impairment (sMCI), we employed a Support Vector Machine (SVM) with a radial basis function (RBF) kernel. This classification technique was executed using a robust 10-fold cross-validation approach, a well-established practice in machine learning evaluation. The radial basis function (RBF) kernel function was chosen due to its universal applicability to various sample distributions. It offers flexibility by adjusting parameters to adapt to the data’s inherent characteristics [80].

We employed a comprehensive set of evaluation metrics to assess the classifier’s performance. These metrics include accuracy (Acc.), sensitivity (Sen.), and specificity (Spec.), which provide insightful information about the classifier’s effectiveness in correctly classifying subjects. The evaluation process involves segregating true labels from the test set, followed by utilizing the trained classifier to predict labels in the test set. The parameters are calculated using the following equations:

(10) $$\text{Accuracy}\;\text{or}\;\text{Acc}\;=\frac{TP+TN}{TP+TN+FP+FN}$$

(11) $$\text{Sensitivity}\;\text{or}\;\text{Sen}\;=\frac{TP}{TP+FN}$$

(12) $$\text{Specificity}\;\text{or}\;\text{Spec}\;=\frac{TN}{TN+FP}$$

where TP, FP, TN, and FN represent true positives, false positives, true negatives, and false negatives, respectively. Here, TP stands for true positives, FP for false positives, TN for true negatives, and FN for false negatives. These metrics collectively illuminate the classifier’s ability to distinguish between pMCI and sMCI subjects.

Additionally, Receiver Operating Characteristic (ROC) curves were employed to visually compare the performance of different classifiers. The ROC curve is a graphical representation where the vertical and horizontal axes represent the false positive rate and true positive rate, respectively. A higher area under the ROC curve indicates superior classifier performance, reflecting its capacity to discriminate between the two classes.

The rigorous evaluation process, encompassing a combination of metrics and visualization techniques, provides a comprehensive assessment of the classification model’s accuracy and reliability in identifying progressive and stable mild cognitive impairment subjects.

## Results

This study employed two distinct methodologies to explore the impact of frequency bands and diverse feature types on classification performance, with feature selection carried out using both PSO and SA algorithms. The outcomes of these investigations are presented in Table 2.

In the first approach, four groups of features were separately used to classify sMCI and pMCI. These groups were 1) all features (100), 2) Graph features (36), 3) MST features (28), and 4) Triads, TMH, and Links features (36) (the number inside the parenthesis indicates the number of features). In this approach, the effect of features over all frequency bands of the graph was studied. The frequency bands were FFB, LFB, MFB, and HFB. In the second approach, features extracted from FFB, LFB, MFB, and HFB were separately used for classification purposes. This analysis could show which frequency bands offered the best and the worst features for classification. For each frequency band, all features of the graph, MST, Triads, TMH, and Links were employed (25 features in total). After feature selection and classification, the results of Table 2 and Table 3 were attained for the first and second approaches, respectively.

By reviewing the results of Table 2, it can be seen that Graph featured offered the highest accuracies for both SA (76%) and PSO (77%) methods. However, the PSO offered 1% and 5% more accuracy and specificity than SA by selecting a much lower number of features (5 features) than SA (17 features). The 5 features, selected by PSO, were (MCC, MS)/Radius/(ME, Modularity) from LFB/MFB/HFB, respectively. The results of using all features are close to those of using Graph features in terms of accuracy, specificity, and sensitivity. However, this closeness was obtained at the cost of using much more features (26 and 55 features for PSO and SA methods, respectively). The least number of selected features were for MST features when using PSO as a feature selection method. In this case, the radius (in FFB) and diameter (in HFB) features offered 72%, 70%, and 75% of accuracy, sensitivity, and specificity, respectively. The lowest classification performance was given by MST features (accuracy of 72%). By reviewing Table 2, it can be said that PSO compared to SA selected much fewer features in many cases while offering similar or better classification performance.

By reviewing the results of the second approach, it is obvious that the worst/the best classification performance was for features of LFB and MFB/ HFB, respectively. The best accuracies in the (LFB and MFB)/HFB were (64% and 63%)/75%, respectively. The best performance in the HFB was offered using 7 features selected by PSO. These 7 features were (GE, ME, AC, MEC) of Graph and (TMHP, |P|, |T2|) of Triads and TMH and Links. The corresponding sensitivity and specificity were 70% and 79%, respectively. In the MFB, an accuracy of 63% was obtained using only one feature (|T2|) selected by SA. Overall, it can be said that the best classification performance in terms of offering higher accuracy with a lower number of features was offered by graph features selected by PSO.

## Discussion

This study presents a novel approach for the classification of stable MCI (sMCI) and progressive MCI (pMCI) using a combination of graph frequency bands and functional connectivity-based features extracted from rs-fMRI data. The classification task was facilitated by employing particle swarm optimization (PSO) and simulated annealing (SA) algorithms for feature selection, followed by support vector machine (SVM) with radial basis function (RBF) kernel for classification. The proposed method aims to predict the conversion of MCI to Alzheimer’s disease (AD) and offers potential insights into the underlying neurobiological changes associated with disease progression.

The research methodology involved several key steps. First, rs-fMRI data preprocessing was conducted using the CONN toolbox, which included various processing steps to ensure data quality and reliability. Functional connectivity (FC) matrices were computed for different frequency bands, namely full-frequency band (FFB), low-frequency band (LFB), middle-frequency band (MFB), and high-frequency band (HFB). These FC matrices were used to extract a diverse set of features, encompassing global graph metrics, minimum spanning tree (MST) metrics, triadic interaction metrics, tendency to make a hub (TMH) metrics, and the number of positive and negative links.

The feature selection process played a crucial role in enhancing classification accuracy. Both PSO and SA algorithms were employed to identify the most relevant features for distinguishing between sMCI and pMCI groups. The resulting feature subsets demonstrated distinct patterns depending on the algorithm used and the type of features considered.

The classification performance of the proposed method was evaluated using a SVM with RBF kernel and a 10-fold cross-validation setup. The results revealed promising accuracy rates, with PSO achieving a 77% accuracy using only 5 selected features. These findings demonstrate the potential clinical utility of the proposed approach for predicting MCI-to-AD conversion, which could inform treatment plans and clinical trials.

Interpretability emerged as a significant advantage of the proposed method, especially in contrast to complex models like deep neural networks (DNNs) that often lack transparency. The selected features in this study were based on the connectivity patterns of distinct brain regions, contributing to a better understanding of the underlying neurobiology.

Key findings from the analysis highlighted the importance of certain features in classification. For instance, ME and Modularity of the HFB were found to be particularly altered between sMCI and pMCI patients, while MCC and MS features of the LFB exhibited strong discriminatory power. Additionally, the radius feature in the MFB was identified as a key contributor to the classification of the two groups.

Comparing the performance of PSO and SA algorithms, PSO stood out by achieving higher accuracy with a smaller number of features. The study underscored the potential of graph analysis of functional connectivity and the effectiveness of the PSO algorithm combined with a simple SVM for accurate classification.

In summary, this study contributes to the field of neuroimaging and cognitive health by presenting a novel approach that combines graph frequency bands, functional connectivity-based features, and advanced feature selection techniques for the classification of stable and progressive MCI. The research addresses the pressing need for early and accurate detection of cognitive decline, particularly in the context of predicting MCI-to-AD conversion.

A notable strength of this study lies in its innovative approach to feature selection. The PSO and SA algorithms effectively navigate the high-dimensional feature space to identify a subset of features that are most relevant for accurate classification. This process enhances model performance, simplifies the classification task, and contributes to interpretability. The selected features shed light on specific connectivity patterns that differentiate sMCI from pMCI patients, offering valuable insights into the neurobiological underpinnings of disease progression.

The study’s findings highlight the importance of different frequency bands and specific connectivity features for classification. The identification of key features, such as Modularity and Mean Eccentricity in the HFB, Mean Clustering Coefficient and Mean Strength in the LFB, and Radius in the MFB, provides meaningful insights into the altered network properties associated with cognitive decline.

The study’s scope focused on the classification of sMCI and pMCI using rs-fMRI data, and future research could extend this framework to larger and more diverse datasets, encompassing longitudinal data to capture temporal changes in connectivity patterns.

In conclusion, this study presents a comprehensive and innovative method for the early classification of stable and progressive MCI using graph frequency bands and functional connectivity-based features. The combination of advanced feature selection techniques and a well-designed classification pipeline demonstrates the potential for accurate prediction of MCI-to-AD conversion. This approach holds promise as a valuable tool for clinicians and researchers seeking to enhance early detection and intervention strategies for neurodegenerative diseases. Continued development and validation of such methodologies have the potential to make a significant impact on the field of cognitive health and the understanding of neurodegenerative processes.

## Availability of data

The data utilized in this study can be accessed from the Alzheimer’s Disease Neuroimaging Institute (ADNI) at http://adni.loni.usc.edu/. Other researchers can access to this data using the same procedures as the authors did. Researchers can access the data by logging into the ADNI website and following these steps: Download > Image Collections > Advanced Search > Search > Select the scans > Add to collection > CSV download > Advanced download. A complete listing of ADNI investigators can be found at: http://adni.loni.usc.edu/study-design/ongoinginvestigations/. The public access to the database is open.

## Figures and Tables

**Table 1.** Sample characteristics.

	sMCI	pMCI
N	142	136
Female (n [%])	59 [41]	68 [50]
Age (mean [SD])	71.75 [8.19]	72.16 [7.83]
MMSE (mean [SD])	26.63 [3.33]	22.94 [3.55]
CDR	0.5	0.5 or 1

CDR: clinical dementia rating, MMSE: mini-mental state exam, pMCI: Progressive mild cognitive impairment, sMCI: stable mild cognitive impairment.

**Table 2.** The results of feature selection. The most important features selected by PSO and SA methods when using Triad, TMH, and Links, Graph, MST, and all features, separately, to classify the sMCI and pMCI subjects.

Frequency band	Selection method	Acc	Sen	Spec	Number of Extracted Features	Number of Selected Features	Selected features	Feature type
**FFB**	PSO	70%	67%	72%	25	5	Diameter, AC	Graph
							Diameter, Kappa, LF	MST
	SA	71%	66%	74%	25	10	MEC, Diameter	Graph
							Radius, Kappa, Deg_max_, BC_max_	MST
							|T_3_|, |P|, |T_2_|, TMH_N_	Triads, TMH, and Links
**HFB**	PSO	75%	70%	79%	25	7	GE, ME, AC, MEC	Graph
							TMH_P_, |P|, |T_2_|	Triads, TMH, and Links
	SA	74%	67%	80%	25	10	MS, MEC, AC, Modularity, ME	Graph
							Diameter	MST
							|N|, TMH_P_, |T_2_|, TMH_N_	Triads, TMH, and Links
**MFB**	PSO	63%	51%	73%	25	6	MCC	Graph
							Deg_max_, T_H_	MST
							|T_3_|, TMH_N_, T_0_	Triads, TMH, and Links
	SA	63%	62%	64%	25	1	|T_2_|	Triads, TMH, and Links
**LFB**	PSO	63%	66%	61%	25	5	T_H_, Deg_max_	MST
							|T_0_|, |T_1_|, |T_2_|	Triads, TMH, and Links
	SA	64%	66%	63%	25	19	GE, Diameter, Modularity, Radius, ME, MCC, MEC	Graph
							Diameter, Radius, Deg_max_, Kappa, T_H_	MST
							Un, |T_1_|, |P|, |N|, AC, TMH_N_	Triads, TMH, and Links

**Table 3.** The results of feature selection. The most important features selected by PSO and SA methods when using features of LFB, MFB, HFB, and FFB, separately, to classify the sMCI and pMCI subjects.

Features	Selection Method	Acc	Sen	Spec	Number of Extracted Features	Number of Selected Features	Selected Features	Frequency Band
**All features**	PSO	75%	70%	80%	100	26	|N|, |P|, BC_max_	FFB
							Radius, ME, |T_1_|, Un, TMH_N_, |T_3_|, MCC	HFB
							|T_2_|, MCC, Radius, Radius (MST), AC, |P|, TMH_P_	MFB
							Diameter, Diameter (MST), TMH_N_, Kappa, |T_3_|, Radius, GE, T_H_, |N|	LFB
	SA	75%	71%	79%	100	55	MS, MEC, ME, Modularity, Radius, |T1|, MCC, LF, |T_3_|, Diameter, AC, |P|	FFB
							MCC, |P|, TMH_P_, Diameter, ME, TMH_N_, Un, T_H_, Radius. Radius (MST), |T_0_|, LF	HFB
							Kappa, MS, TMH_N_, |T_3_|, BC_max_, T_H_, Radius, |N|, |T_2_|, ME, Deg_max_, Diameter, GE, Diameter (MST), P	MFB
							Diameter, MCC, |T_0_|, |T_2_|, Deg_max_, MEC, Modularity, |T_3_|, T_H_, Radius, MS, Kappa, |N|, Un	LFB
**Graph features**	**PSO**	**77%**	**70%**	**83%**	**36**	**5**	**ME, Modularity**	**HFB**
							**Radius**	**MFB**
							**MCC, MS**	**LFB**
	SA	76%	73%	78%	36	17	MS, Diameter, ME, Modularity	FFB
							MS, ME, Radius, MEC, GE	HFB
							ME, Modularity, AC, MCC, MEC	MFB
							Diameter, Radius. MCC, AC	LFB
**MST features**	PSO	72%	70%	75%	28	2	Radius	FFB
							Diameter	HFB
	SA	72%	71%	72%	28	8	Radius, Diameter	FFB
							Diameter, T_H_	HFB
							Kappa	MFB
							BC_max_, T_H_	LFB
**Triads, TMH, and Links features**	PSO	74%	65%	82%	36	12	|T_3_|, TMH_P_	FFB
							Un, |P|, TMH_N_, |T_1_|	HFB
							TMH_N_, |T_3_|, |T_0_|, TMH_P_	MFB
							|T_1_|, TMH_N_	LFB
	SA	73%	64%	81%	36	5	TMH_P_	FFB
							TMH_P_, TMH_N_	HFB
							|T_2_|	MFB
							|N|	LFB

## Key Points:

1. Graph frequency band filters offer functional data with discriminative features between stable MCI (sMCI) and progressive MCI (pMCI).
2. Global metrics of graph yield superior classification performance, requiring only a modest subset of five features for optimal discrimination.
3. Integration of functional connectivity-based features promises to enhance prediction capabilities for individuals with MCI, aiding in early diagnosis and intervention.

## References

[1] Peterson RC, Smith GE, Waring SC, Ivnik RJ, Tangalos EG, Kokmen E. Mild cognitive impairment: clinical characterization and outcome. Arch Neurol 1999; 56:303–8. 10190820 10.1001/archneur.56.3.303

[2] Petersen RC, Roberts RO, Mild Cognitive Impairment: Ten Years Later. Arch Neurol. 2009; 66:1447–55. 20008648 10.1001/archneurol.2009.266 PMC3081688

[3] Spillantini MG, Goedert M. Tau pathology and neurodegeneration. The Lancet Neurology. 2013 Jun 1; 12(6):609–22. 23684085 10.1016/S1474-4422(13)70090-5

[4] Bateman RJ, Xiong C, Benzinger TL, Fagan AM, Goate A, Fox NC, Marcus DS, Cairns NJ, Xie X, Blazey TM, Holtzman DM. Clinical and biomarker changes in dominantly inherited Alzheimer’s disease. N Engl J Med. 2012 Aug 30; 367:795–804. 22784036 10.1056/NEJMoa1202753 PMC3474597

[5] Shakil S., Lee C. H., and Keilholz S. D. (2016). Evaluation of sliding window correlation performance for characterizing dynamic functional connectivity and brain states. Neuroimage 133, 111–128. doi: 10.1016/j.neuroimage.2016.02.074. 26952197 PMC4889509

[6] Khazaee A., Ebrahimzadeh A., and Babajani-Feremi A. (2015). Identifying patients with Alzheimer’s disease using resting-state fMRI and graph theory. Clin. Neurophysiol. Offic. 126, 2132–2141. doi: 10.1016/j.clinph.2015.02.060. 25907414

[7] Allen EA, Damaraju E, Plis SM, Erhardt EB, Eichele T, Calhoun VD. Tracking whole-brain connectivity dynamics in the resting state. Cerebral cortex. 2014 Mar 1;24(3):663–76. 23146964 10.1093/cercor/bhs352 PMC3920766

[8] Chang C, Glover GH. Time–frequency dynamics of resting-state brain connectivity measured with fMRI. Neuroimage. 2010 Mar 1;50(1):81–98. 20006716 10.1016/j.neuroimage.2009.12.011 PMC2827259

[9] Hinrichs C, Singh V, Xu G, Johnson SC, ADNI. Predictive markers for AD in a multi-modality framework: an analysis of MCI progression in the ADNI population. Neuroimage 2011; 55:574–89. 21146621 10.1016/j.neuroimage.2010.10.081 PMC3035743

[10] Young J, Modat M, Cardoso MJ, Mendelson A, Cash D, Ourselin S, Accurate multimodal probabilistic prediction of conversion to Alzheimer’s disease in patients with mild cognitive impairment. Neuroimage Clin 2013; 2:735–45. 24179825 10.1016/j.nicl.2013.05.004 PMC3777690

[11] Liu Y, Mattila J, Ruiz MAM, Paajanen T, Koikkalainen J, van Gils M, and Predicting AD conversion: comparison between prodromal AD guidelines and computer assisted predict AD tool. PLoS One 2013; 8:e55246. 23424625 10.1371/journal.pone.0055246 PMC3570420

[12] Zamani J., Sadr A., & Javadi A. H. (2022). Classification of early-MCI patients from healthy controls using evolutionary optimization of graph measures of resting-state fMRI, for the Alzheimer’s disease neuroimaging initiative. PloS one, 17(6), e0267608. 35727837 10.1371/journal.pone.0267608 PMC9212187

[13] Yassa M. A. High-resolution structural and functional MRI of hippocampal CA3 and dentate gyrus in patients with amnestic Mild Cognitive Impairment. NeuroImage 51, 1242–1252 (2010). 20338246 10.1016/j.neuroimage.2010.03.040 PMC2909476

[14] Sperling R. The potential of functional MRI as a biomarker in early Alzheimer’s disease. Neurobiology of Aging 32, S37–S43 (2011). 22078171 10.1016/j.neurobiolaging.2011.09.009 PMC3233699

[15] Wierenga C. E. & Bondi M. W. Use of functional magnetic resonance imaging in the early identification of Alzheimer’s disease. Neuropsychology Review 17, 127–143 (2007). 17476598 10.1007/s11065-007-9025-y PMC2084460

[16] Pievani M., de Haan W., Wu T., Seeley W. W. & Frisoni G. B. Functional network disruption in the degenerative dementias. The Lancet Neurology 10, 829–843 (2011). 21778116 10.1016/S1474-4422(11)70158-2 PMC3219874

[17] Teipel S. Multimodal imaging in Alzheimer’s disease: Validity and usefulness for early detection. The Lancet Neurology 14, 1037–1053 (2015). 26318837 10.1016/S1474-4422(15)00093-9

[18] Lee M. H., Smyser C. D. & Shimony J. S. Resting-state fMRI: A review of methods and clinical applications. American Journal of Neuroradiology 34, 1866–1872 (2013). 22936095 10.3174/ajnr.A3263 PMC4035703

[19] Vemuri P., Jones D. T. & Jack C. R. Resting state functional MRI in Alzheimer’s disease. Alzheimer’s Research and Therapy 4, 1–9 (2012). 10.1186/alzrt100 PMC3471422 22236691

[20] Fox M. D. & Greicius M. Clinical applications of resting state functional connectivity. Frontiers in Systems Neuroscience 4, (2010). 10.3389/fnsys.2010.00019 PMC2893721 20592951

[21] Greicius M. D., Krasnow B., Reiss A. L. & Menon V. Functional connectivity in the resting brain: A network analysis of the default mode hypothesis. Proceedings of the National Academy of Sciences of the United States of America 100, 253–258 (2003). 12506194 10.1073/pnas.0135058100 PMC140943

[22] Sheline Y. I. & Raichle M. E. Resting state functional connectivity in preclinical Alzheimer’s disease. Biological Psychiatry 74, 340–347 (2013). 23290495 10.1016/j.biopsych.2012.11.028 PMC3537262

[23] Zhang H. Y. Resting brain connectivity: Changes during the progress of Alzheimer disease. Radiology 256, 598–606 (2010). 20656843 10.1148/radiol.10091701

[24] Zhou J. Divergent network connectivity changes in behavioural variant frontotemporal dementia and Alzheimer’s disease. Brain 133, 1352–1367 (2010). 20410145 10.1093/brain/awq075 PMC2912696

[25] Dennis E. L. & Thompson P. M. Functional brain connectivity using fMRI in aging and Alzheimer’s disease. Neuropsychology Review 24, 49–62 (2014). 24562737 10.1007/s11065-014-9249-6 PMC4109887

[26] Jalilianhasanpour R., Beheshtian E., Sherbaf F. G., Sahraian S. & Sair H. I. Functional Connectivity in Neurodegenerative Disorders: Alzheimer’s Disease and Frontotemporal Dementia. Topics in Magnetic Resonance Imaging 28, 317–324 (2019). 31794504 10.1097/RMR.0000000000000223

[27] Zhan Y. Longitudinal Study of Impaired Intra- and Inter-Network Brain Connectivity in Subjects at High Risk for Alzheimer’s Disease. Journal of Alzheimer’s Disease 52, 913–927 (2016). 10.3233/JAD-160008 27060962

[28] Bullmore E. & Sporns O. Complex brain networks: Graph theoretical analysis of structural and functional systems. Nature Reviews Neuroscience 10, 186–198 (2009). 19190637 10.1038/nrn2575

[29] Rubinov M. & Sporns O. Complex network measures of brain connectivity: Uses and interpretations. NeuroImage 52, 1059–1069 (2010). 19819337 10.1016/j.neuroimage.2009.10.003

[30] van den Heuvel M. P. & Sporns O. Network hubs in the human brain. Trends in Cognitive Sciences 17, 683–696 (2013). 24231140 10.1016/j.tics.2013.09.012

[31] Farahani F. v., Karwowski W. & Lighthall N. R. Application of graph theory for identifying connectivity patterns in human brain networks: A systematic review. Frontiers in Neuroscience 13, 1–27 (2019). 31249501 10.3389/fnins.2019.00585 PMC6582769

[32] Blanken T. F. Connecting brain and behavior in clinical neuroscience: A network approach. Neuroscience and Biobehavioral Reviews 130, 81–90 (2021). 34324918 10.1016/j.neubiorev.2021.07.027

[33] Yun J. Y. & Kim Y. K. Graph theory approach for the structural-functional brain connectome of depression. Progress in Neuro-Psychopharmacology and Biological Psychiatry 111, 110401 (2021). 34265367 10.1016/j.pnpbp.2021.110401

[34] Amiri S., Arbabi M., Kazemi K., Parvaresh-Rizi M. & Mirbagheri M. M. Characterization of brain functional connectivity in treatment-resistant depression. Progress in Neuro-Psychopharmacology and Biological Psychiatry 111, 110346 (2021). 33961964 10.1016/j.pnpbp.2021.110346

[35] Beheshti I. & Ko J. H. Modulating brain networks associated with cognitive deficits in Parkinson’s disease. Molecular Medicine 27, (2021). 10.1186/s10020-021-00284-5 PMC7945662 33691622

[36] Dai Z. Identifying and mapping connectivity patterns of brain network hubs in Alzheimer’s disease. Cerebral Cortex 25, 3723–3742 (2015). 25331602 10.1093/cercor/bhu246

[37] Tijms B. M. Alzheimer’s disease: connecting findings from graph theoretical studies of brain networks. Neurobiology of Aging 34, 2023–2036 (2013). 23541878 10.1016/j.neurobiolaging.2013.02.020

[38] Brier M. R. Functional connectivity and graph theory in preclinical Alzheimer’s disease. Neurobiology of Aging 35, 757–768 (2014). 24216223 10.1016/j.neurobiolaging.2013.10.081 PMC3880636

[39] He Y. & Evans A. Graph theoretical modeling of brain connectivity. Current Opinion in Neurology 23, 341–350 (2010). 20581686 10.1097/WCO.0b013e32833aa567

[40] Bassett D. S. & Bullmore E. T. Human brain networks in health and disease. Current Opinion in Neurology 22, 340–347 (2009). 19494774 10.1097/WCO.0b013e32832d93dd PMC2902726

[41] Stam C. J. Modern network science of neurological disorders. Nature Reviews Neuroscience 15, 683–695 (2014). 25186238 10.1038/nrn3801

[42] Hojjati S. H., Ebrahimzadeh A., Khazaee A. & Babajani-Feremi A. Predicting conversion from MCI to AD using resting-state fMRI, graph theoretical approach and SVM. Journal of Neuroscience Methods 282, 69–80 (2017). 28286064 10.1016/j.jneumeth.2017.03.006

[43] Khazaee A., Ebrahimzadeh A. & Babajani-Feremi A. Classification of patients with MCI and AD from healthy controls using directed graph measures of resting-state fMRI. Behavioural Brain Research 322, 339–350 (2017). 27345822 10.1016/j.bbr.2016.06.043

[44] Behfar Q. Graph theory analysis reveals resting-state compensatory mechanisms in healthy aging and prodromal Alzheimer’s disease. Frontiers in Aging Neuroscience 12, 1–13 (2020). 33192468 10.3389/fnagi.2020.576627 PMC7642892

[45] Gregory S. Operationalizing compensation over time in neurodegenerative disease. Brain 140, 1158–1165 (2017). 28334888 10.1093/brain/awx022 PMC5382953

[46] Yao Z. Abnormal cortical networks in mild cognitive impairment and alzheimer’s disease. PLoS Computational Biology 6, (2010). 10.1371/journal.pcbi.1001006 PMC2987916 21124954

[47] Cabeza R. Maintenance, reserve and compensation: the cognitive neuroscience of healthy ageing. Nature Reviews Neuroscience 19, 701–710 (2018). 10.1038/s41583-018-0068-2 PMC6472256 30305711

[48] Guo H., Liu L., Chen J., Xu Y., & Jie X. (2017). Alzheimer classification using a minimum spanning tree of high-order functional network on fMRI dataset. Frontiers in neuroscience, 11, 639. 29249926 10.3389/fnins.2017.00639 PMC5717514

[49] van Dellen E., Sommer I. E., Bohlken M. M., Tewarie P., Draaisma L., Zalesky A., … & Stam C. J. (2018). Minimum spanning tree analysis of the human connectome. Human brain mapping, 39(6), 2455–2471. 29468769 10.1002/hbm.24014 PMC5969238

[50] Moradimanesh Z., Khosrowabadi R., Gordji M. E., & Jafari G. R. 2021. Altered structural balance of resting-state networks in autism. Scientific reports, 11(1), 1–16. 33479287 10.1038/s41598-020-80330-0 PMC7820028

[51] Saberi M., Khosrowabadi R., Khatibi A., Misic B., Jafari G. 2021. Topological impact of negative links on the stability of resting-state brain network. Scientific reports, 11(1), 1–14. 33500525 10.1038/s41598-021-81767-7 PMC7838299

[52] Shuman D. I., Narang S. K., Frossard P., Ortega A., Vandergheynst P., 2013. The emerging field of signal processing on graphs: Extending high dimensional data analysis to networks and other irregular domains. IEEE Signal Processing Magazine, 30, 83–98.

[53] Ortega A., Frossard P., Kovačević J., Moura J. M., Vandergheynst P., 2018. Graph signal processing: Overview, challenges, and applications. Proceedings of the IEEE, 106(5), 808–828.

[54] Jafadideh A. T., & Asl B. M. 2022. Rest-fMRI based comparison study between autism spectrum disorder and typically control using graph frequency bands. Computers in Biology and Medicine, 105643. 35598352 10.1016/j.compbiomed.2022.105643

[55] Jafadideh A. T., Asl B. M. 2021. A Comparison Study between Autism Spectrum Disorder and Typically Control in Graph Frequency Bands Using Graph and Triadic Interaction Metrics. bioRxiv.

[56] Jafadideh A. T., & Asl B. M. (2022). Rest-fMRI based comparison study between autism spectrum disorder and typically control using graph frequency bands. Computers in Biology and Medicine, 146, 105643. 35598352 10.1016/j.compbiomed.2022.105643

[57] Talesh Jafadideh A., & Mohammadzadeh Asl B. (2022). Structural filtering of functional data offered discriminative features for autism spectrum disorder. Plos one, 17(12), e0277989. 36472989 10.1371/journal.pone.0277989 PMC9725140

[58] Padole H. P. (2021). Some studies on graph signal processing with application to alzheimer’s disease detection (Doctoral dissertation, IIT Delhi).

[59] Fan Y., Batmanghelich N., Clark C.M., Davatzikos C., Spatial patterns of brain atrophy in MCI patients, identified via high-dimensional pattern classification, predict subsequent cognitive decline, Neuroimage 39 (4) (2008) 1731–1743. 18053747 10.1016/j.neuroimage.2007.10.031 PMC2861339

[60] Cabral C, Morgado PM, Campos Costa D, Silveira M; Alzheimer׳s Disease Neuroimaging Initiative. Predicting conversion from MCI to AD with FDG-PET brain images at different prodromal stages. Comput Biol Med. 2015 Mar; 58:101–9. doi:10.1016/j.compbiomed.2015.01.003. Epub 2015 Jan 12. 25625698

[61] Blum A. L. & Langley P. Selection of relevant features and examples in machine learning. Artificial Intelligence 97, 245–271 (1997).

[62] Reunanen J. Overfitting in making comparisons between variable selection methods. Journal of Machine Learning Research 3, 1371–1382 (2003).

[63] John G. H., Kohavi R. & Pfleger K. Irrelevant Features and the Subset Selection Problem. in Machine Learning Proceedings 1994 121–129 (Elsevier, 1994). doi:10.1016/B978-1-55860-335-6.50023-4.

[64] Chu C., Hsu A. L., Chou K. H., Bandettini P. & Lin C. P. Does feature selection improve classification accuracy? Impact of sample size and feature selection on classification using anatomical magnetic resonance images. NeuroImage 60, 59–70 (2012). 22166797 10.1016/j.neuroimage.2011.11.066

[65] Bicacro E., Silveira M., Marques J.S., Alternative feature extraction methods in 3D brain image-based diagnosis of Alzheimer’s disease, in: 19th IEEE International Conference on Image Processing (ICIP), IEEE, 2012, pp. 1237–1240.

[66] Mann H. B., Whitney D. R. 1947. On a test of whether one of two random variables is stochastically larger than the other. The annals of mathematical statistics, 50–60.

[67] Abualigah L. M., Khader A. T., & Hanandeh E. S. (2018). A new feature selection method to improve the document clustering using particle swarm optimization algorithm. Journal of Computational Science, 25, 456–466.

[68] Mafarja M. M., & Mirjalili S. (2017). Hybrid whale optimization algorithm with simulated annealing for feature selection. Neurocomputing, 260, 302–312.

[69] Jack C.R., Bernstein M.A., Fox N.C., Thompson P., Alexander G., Harvey D., Borowski B., Britson P.J., L. Whitwell J., Ward C., Dale A.M., Felmlee J.P., Gunter J.L., Hill D.L.G., Killiany R., Schuff N., Fox-Bosetti S., Lin C., Studholme C., DeCarli C.S., Gunnar Krueger, Ward H.A., Metzger G.J., Scott K.T., Mallozzi R., Blezek D., Levy J., Debbins J.P., Fleisher A.S., Albert M., Green R., Bartzokis G., Glover G., Mugler J., Weiner M.W., 2008. The Alzheimer’s disease neuroimaging initiative (ADNI): MRI methods. J. Magn. Reson. Imaging 27, 685–691. 10.1002/jmri.21049. 18302232 PMC2544629

[70] Jack C.R., Bernstein M.A., Borowski B.J., Gunter J.L., Fox N.C., Thompson P.M., Schuff N., Krueger G., Killiany R.J., DeCarli C.S., Dale A.M., Carmichael O.W., Tosun D., Weiner M.W., 2010a. Updateon the Magnetic Resonance Imaging core of the Alzheimer’s disease Neuroimaging Initiative. Alzheimer’s & Dementia 6, 212–220. 10.1016/j.jalz.2010.03.004. PMC2886577 20451869

[71] Whitfield-Gabrieli S., & Nieto-Castanon A. (2012). Conn: a functional connectivity toolbox for correlated and anticorrelated brain networks. Brain connectivity, 2(3), 125–141. 22642651 10.1089/brain.2012.0073

[72] Huang W., Bolton T. A., Medaglia J. D., Bassett D. S., Ribeiro A., Van De Ville D., 2018. A graph signal processing perspective on functional brain imaging. Proceedings of the IEEE, 106(5), 868–885.

[73] Allen E. A., Damaraju E., Plis S. M., Erhardt E. B., Eichele T., Calhoun V. D., 2014. Tracking whole-brain connectivity dynamics in the resting state. Cerebral Cortex, 24(3), 663–676. 23146964 10.1093/cercor/bhs352 PMC3920766

[74] Fornito A., Zalesky A., Bullmore E. 2016. Fundamentals of brain network analysis. Academic Press.

[75] Rubinov M., Sporns O. 2010. Complex network measures of brain connectivity uses and interpretations. Neuroimage, 52(3), 1059–1069. 19819337 10.1016/j.neuroimage.2009.10.003

[76] Van Mieghem P., Magdalena S. M. 2005. Phase transition in the link weight structure of networks. Physical Review E, 72(5), 056138. 10.1103/PhysRevE.72.056138 16383719

[77] Guo H., Liu L., Chen J., Xu Y., Jie X. 2017. Alzheimer classification using a minimum spanning tree of high-order functional network on fMRI dataset. Frontiers in neuroscience, 11, 639. 29249926 10.3389/fnins.2017.00639 PMC5717514

[78] Lee H., Kang H., Chung M. K., Kim B. N., Lee D. S. 2012. Persistent brain network homology from the perspective of dendrogram. IEEE transactions on medical imaging, 31(12), 2267–2277. 23008247 10.1109/TMI.2012.2219590

[79] Liu X., Yang H., Becker B., Huang X., Luo C., Meng C., Biswal B. 2021. Disentangling age-and disease-related alterations in schizophrenia brain network using structural equation modeling: A graph theoretical study based on minimum spanning tree. Human brain mapping, 42(10), 3023–3041. 33960579 10.1002/hbm.25403 PMC8193510

[80] Noble W. What is a support vector machine?. Nat Biotechnol 24, 1565–1567 (2006). 17160063 10.1038/nbt1206-1565

